# Designing greener participant-centred trials: an analysis of ‘carbon relevant’ factors within items that influence participants’ decisions about trial recruitment and retention

**DOI:** 10.1186/s13063-024-08083-z

**Published:** 2024-04-15

**Authors:** Emilia Piltonen, Beatriz Goulao, Katie Gillies

**Affiliations:** Health Services Research Unit, University of Aberdeen, Aberdeen, UK

## Introduction

The World Health Organization have recognised climate change as one of the biggest health threats facing humanity, considering its effects on social and environmental determining factors of health such as clean air, potable water, adequate food and secure shelter [1]. In 2007, the Sustainable Trials Study Group concluded that ‘clinical trials contribute to greenhouse gas emissions, and estimates in 2023 predicted that the 350,000 trials registered on ClinicalTrials.gov have a carbon usage of 27.5 M tonnes [2, 3]. Nevertheless, clinical trials are essential for identifying effective and safe treatments and preventing diseases. General recommendations targeting the reduction of carbon usage in research (National Institute for Health Research (NIHR) Carbon Reduction Guidelines) were published in 2019 yet progress to decarbonise trials remains slow [3, 4]. Of the small number of studies that have explored carbon usage in clinical trials, factors such as energy usage in research premises, trial team, and participant-related travel have been identified as key impacts [5, 6].

Efforts to deliver more environmentally sustainable research, including clinical trials, continue to gather pace both in academic and industry-led trials, in line with the acute growth of the climate threat worldwide [6]. A recent paper by Griffiths et al. developed a method and associated guidance to help trialists to efficiently estimate the carbon footprint of a clinical trial and, consequently, increase the trialists’ recognition of trial-specific carbon emissions and ability to enhance climate change mitigation [3]. One of the key considerations for clinical trials going forwards is to consider the trade-offs in design aspects with rigour and patient acceptability, e.g. reducing the number of in-person visits for follow-up to reduce the carbon footprint balanced against impacts on trial retention [6]. There is currently very little evidence as to what or whether carbon-relevant aspects of trials (e.g. patient travel, mode of data collection) influence patients’ decision-making in relation to trial participation. The findings outlined in this letter explore which ‘carbon relevant’ factors are related to previously identified factors influencing patients’ decision-making in relation to taking part (recruitment) and staying (retention) in a clinical trial.

## Methods

### Data collection

A secondary analysis of findings reported in existing qualitative evidence syntheses (QES) that reported the influences on patients’ decisions about taking part (recruitment) or staying in (retention) a clinical trial was used as the data set to be coded [7, 8]. The findings from the original reviews did not report impacts on climate change these inferences were made during the secondary analysis. These reviews were chosen as they were both published within the last 5 years and provide summary data of the main influences on patients in relation to recruitment and retention across a number of trials in a range of settings and therefore should offer transferable relevant learning. These reviews were a convenience sample known to the study team and a formal search was not conducted.

### Data extraction

The themes from the qualitative evidence syntheses were extracted verbatim from the published results and collated into an Excel spreadsheet. The verbatim themes were extracted first, followed by subthemes, and then an individual item-level finding. Additionally, a fourth column provided quotes obtained from the published QES to add further explanation and context to the themes/sub-themes/individual item findings. Following data extraction of the patient-reported influences on trial recruitment and retention, a list of potential carbon-relevant factors was drafted. The carbon-relevant factors were taken directly from a recent paper describing a method to quantify the carbon footprint of clinical trials [3] and include the following:

1. Trial setup: productions, provision and postage of documentation to sites and/or patients
2. CTU emissions: energy consumption, heating and commuting for the main trial team
3. Trial-specific meetings and travel: trial staff travel, sustenance and hotel stays
4. Intervention: processes related to providing and delivering the trial intervention that are over and above routine care
5. Data collection and exchange: how it was collected and stored, etc.
6. Trial supplies and equipment: equipment used by the trial team, sites and/or participants
7. Trial-specific patient assessments: study assessments
8. Samples: collecting samples using sample kits and/or blood samples
9. Laboratory: laboratory staff
10. Trial close out: storage of samples and documentation after the trial ends

### Data analysis

The patient recruitment and retention themes were presented in individual rows of the Excel spreadsheet and the carbon-relevant factors in columns. Directed content analysis was used to analyse the data extracted from the evidence syntheses [9]. Data that was relevant for a particular trial theme (i.e. patient recruitment or patient retention) was coded against the predetermined carbon-relevant codes. We were generous in our coding, and if the reported individual item could have a broadly applicable carbon-relevant impact, it was included as an influence. For example, an individual item reported as relevant for recruitment and retention that may require equipment used by CTUs would be coded against carbon factor, ‘trial supplies and equipment’. This could include the use of electronic equipment used to produce a written invitation letter. The carbon-relevant factors for both recruitment and retention findings were coded independently by all three team members and then discussed for agreement on clarity/relevance. Data was summarised using counts and percentages.

## Results

### Included study characteristics

The first of the evidence sources included a review of participant-reported influences on recruitment to trials and included 29 studies reporting data from 847 potential participants. The studies included those who had consented to take part in a trial (*n* = 10), those who had declined (*n* = 7), or both (*n* = 12). The majority of the studies were conducted in the UK (*n* = 16) or Europe (*n* = 6) across various clinical areas with the majority in medicine (*n* = 11) and pharmaceutical interventions being most frequently tested (*n* = 11). The second review explored participants’ reasons for not completing a trial until the end. It included 11 studies with findings from 168 people. Again, the majority of studies were based in the UK (*n* = 5), with a clinical focus on mental health (*n* = 5), and the most frequently tested interventions were psycho-educational and/or cognitive therapy-based tools (*n* = 4). All studies across both reviews included adult participants.

### Patient important carbon relevant influences on recruitment and retention

Additional file 1: Table S1 describes the recruitment themes and specific components within the themes identified and how they map to carbon-relevant factors. From the original 3 themes and 8 subthemes reported in the included review, we identified 27 individual items (e.g. face-to-face communication preferred when giving trial information). Eleven of these individual participant-reported influences on trial recruitment mapped to five of the ten carbon-relevant factors. These five most frequently coded carbon-relevant factors were ‘trial supplies and equipment’ (*n* = 9), followed by ‘CTU emissions’ (*n* = 8), and then ‘trial-specific patient assessment’ (*n* = 7), ‘meetings and travel’ (*n* = 6) and ‘trial setup’ (*n* = 5). The remaining five carbon factors (intervention, data collection and exchange, samples, lab, analysis and trial close out) had 0 participant-reported influences coded. All five carbon factors identified as relevant were coded to items within the theme of ‘trial influence on decision to participate’ with no carbon relevant factors identified for ‘personal influence on decision making’ or ‘the impact of potential outcomes to participate’. The theme of ‘trial influence on decision to participate’ included the following, example, individual items and links to carbon factors:

- 1.1.2. ‘Written communication beneficial to face-to-face trial information’—linked to carbon-relevant factors of production of trial documents, provision/postage of information, CTU emissions, equipment used by CTUs and in some cases attendance at trial-specific patient assessments for consent.
- 1.2.2. ‘Comprehensive’ and ‘extensive’ briefing of trial participation was preferred to participants—linked to carbon-relevant factors of production of trial documents, CTU emissions, equipment used by CTUs, visits to sites for training and visits of participants for ‘extensive briefing’.

Additional file 2: Table S2 describes the retention themes and specific components within the themes identified and how they map to carbon-relevant factors. Out of the five original themes (and 10 subthemes) reported in the included review, 23 individual items were identified (e.g. perceptions of recovery as a reason to not continue trial medication). There were fewer carbon-relevant factors coded against the participant-reported influences on retention than recruitment, with 4 out of the 10 carbon factors identified as relevant. These were ‘interventions’ (*n* = 2), ‘CTU emissions’ (*n* = 1), ‘meetings and travel’ (*n* = 1) and ‘trial supplies and equipment’ (*n* = 1). These factors coded to 2 retention themes: ‘the “it” of aspects of the trial with individual preferences of care and support’ which were all linked to the carbon factor of ‘interventions’ and ‘the compatibility of aspects of trial processes with individual capabilities’. The theme of ‘trial influence on decision to participate’ included the following, example, individual items and links to carbon factors:

- 2.1.2. Interventions being too technical, too physically demanding and too intensive—linked to carbon-relevant factors through utilities required for intervention delivery and activities or resources required relating to intervention delivery
- 3.1.3. Communication and cultural issues—linked to carbon-relevant factors through assumptions that CTU emissions, equipment and travel will increase with the need for translation or to enhance communication

**Fig. 1:**
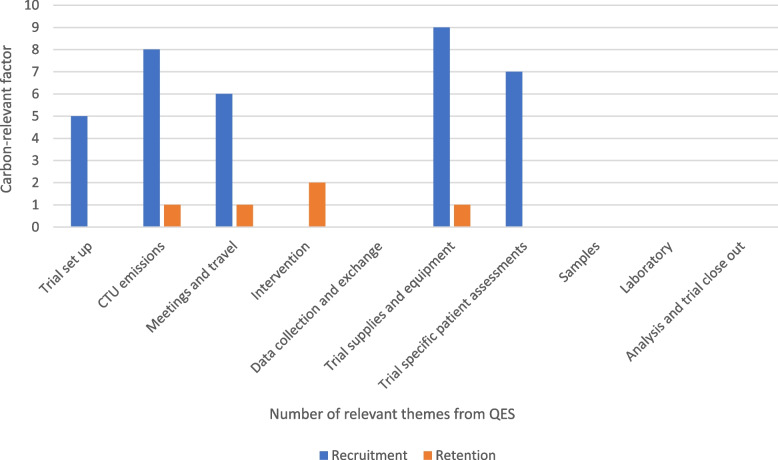
Carbon-relevant factors applicable to participant-reported influences on trial recruitment and retention

Figure 1 provides a graph of the carbon-relevant factors by recruitment and retention influence.

## Discussion

This secondary analysis has identified carbon-relevant factors that are applicable to participant-reported influences on trial recruitment and retention. The most frequently identified carbon-relevant factors in this study are consistent with the most frequent reported in the recent carbon footprinting exercise of two clinical trials, namely CTU emissions and trial-specific patient assessments [3]. This suggests that when making adjustments to these areas to minimize carbon impacts during trial design, the indirect influence on participant recruitment and retention should be considered. Trial supplies and equipment were the most frequently cited carbon-relevant factor across recruitment influences but, interestingly, only made up a much smaller proportion of the carbon contribution in the recent footprinting exercise of two completed trials [3]. The data for influences on retention and the relevance for carbon was more absent in the data set. This highlights one of the key limitations of this work, which the analysis is based on existing qualitative data, where for recruitment it was a sub-set of all identified studies and for retention it was already a relatively small number of studies. There are a few key papers that have been published since the included evidence syntheses were published which may alter some of the findings. For example, studies that have explored specific aspects of return of data collection which would code to the ‘data collection and exchange’ are missing [10, 11]. However, the analysis presented here does begin to go some way to indirectly consider which aspects of trial recruitment and retention that are important to participants are carbon relevant. Future studies should ask participants directly to consider the influence of carbon-relevant factors when exploring the influence on recruitment and retention and explore trade-offs in the design and conduct that would enable the delivery of greener trials. As a minimum, these trade-offs in terms of design and conduct with carbon impacts could also be discussed with patient partners during the design stage of trials in development.

### Supplementary Information

**Additional file 1: Table S1.** Reported influences on participant recruitment mapped to carbon relevant factors.

**Additional file 2: Table S2.** Reported influences on participant retention mapped to carbon relevant factors.

## Data Availability

Secondary analysis of published data.
