# Supplementary material for: Designing greener participant-centred trials: an analysis of ‘carbon relevant’ factors within items that influence participants’ decisions about trial recruitment and retention
Source: Trials. 2024 Apr 15;25:260. doi: 10.1186/s13063-024-08083-z (PMC11017479; doi:10.1186/s13063-024-08083-z)
Supplement: Supplementary file 1 — Additional file 1: Table S1. Reported influences on participant recruitment mapped to carbon relevant factors. [file 13063_2024_8083_MOESM1_ESM.docx]

**Supplementary Table 1 -** – Reported influences on participant recruitment mapped to carbon relevant factors

| **Recruitment theme** | | | **Carbon relevant factors** | | | | | | | | | |  |
| --- | --- | --- | --- | --- | --- | --- | --- | --- | --- | --- | --- | --- | --- |
| **Verbatim theme** | **Sub-theme** | **Individual item** | **1. Trial set up** | **2. CTU emissions** | **3. Meetings and travel** | **4. Interventions** | **5. Data collection and exchange** | **6. Trial supplies and equipment** | **7. Trial specific patient assessment** | **8. Samples** | **9. Laboratory** | **10. Analysis and trial close out** | **Total of themes** |
| 1.Trial influence on decision to participate | 1.1. Communication of trial information | 1.1.1. **Face-to-face communication** preferred when giving trial information |  | **+** | **+** |  |  | **+** | **+** |  |  |  | **4** |
|  |  | 1.1.2. **Written communication** beneficial to face-to-face trial information | **+** | **+** |  |  |  | **+** |  |  |  |  | **3** |
|  |  | 1.1.3. **The person delivering the trial information** should have good communication skills, be knowledgeable, use human and person-centred approach to research | **+** | **+** | **+** |  |  | **+** | **+** |  |  |  | **5** |
|  | 1.2. Quality of trial information | 1.2.1. Good **information provided on the trial** which should include details about options, drug doses, risks, side effects, time commitment, treatment equivalence, randomisation process, etc. |  |  |  |  |  | **+** |  |  |  |  | **1** |
|  |  | 1.2.2. **"Comprehensive" and "extensive" briefing** of trial participation was preferred to participants | **+** | **+** | **+** |  |  | **+** | **+** |  |  |  | **5** |
|  |  | 1.2.3. **Simple & straightforward briefing** decrease risk of confusion or information overload according to non-participants |  |  |  |  |  | **+** |  |  |  |  | **1** |
|  |  | 1.2.4. Be thoughtful of **timing of delivering trial information** to minimize confusion with their standard treatment |  | **+** | **+** |  |  | **+** | **+** |  |  |  | **4** |
|  |  | 1.2.5. Knowledge and understanding of **free to withdraw in randomization trial** minimize confusion of concepts and trial intervention |  |  |  |  |  |  |  |  |  |  | **0** |
|  | 1.3. Trial components | 1.3.1. **Randomization** could negatively effect potential participants' decisions if they had a clear preference for a particular treatment |  |  |  |  |  |  | **+** |  |  |  | **1** |
|  |  | 1.3.2. **Financial reimbursement** is considered as acknowledgement of time and effort, but not sole reason | **+** | **+** |  |  |  | **+** |  |  |  |  | **3** |
|  |  | 1.3.3. **Other incentives** such as additional health checks or medications are regarded as acknowledgement of time and effort | **+** | **+** | **+** |  |  | **+** | **+** |  |  |  | **5** |
|  |  | 1.3.4. **Additional appointments/ travel** may be considered as a burden or disruptive aspect of trial intervention |  | **+** | **+** |  |  |  | **+** |  |  |  | **3** |
| 2. Personal influence on decision making | 2.1. External influences on decision-making | 2.1.1. **Influence from external people** such as family, friends, healthcare professionals and previous trial participants |  |  |  |  |  |  |  |  |  |  | **0** |
|  |  | 2.1.2. **More trust in recruiting physicians they know** than recruiting clinicians they do not know |  |  |  |  |  |  |  |  |  |  | **0** |
|  |  | 2.1.3. Exposure to **internet and media** could be either positive or negative |  |  |  |  |  |  |  |  |  |  | **0** |
|  | 2.2. Risks not worth taking | 2.2.1. **Risk of personal harm** such as feeling like a 'guinea pig' and concerned of taking tested drugs might be considered too risky |  |  |  |  |  |  |  |  |  |  | **0** |
|  |  | 2.2.2. **Considering their health as good or healthy** and not wanting to identify additional health problems might make them deem themselves ineligible |  |  |  |  |  |  |  |  |  |  | **0** |
|  |  | 2.2.3. **Too many health problems** might make one ineligible |  |  |  |  |  |  |  |  |  |  | **0** |
|  | 2.3. Making it worth taking the risk of trial participation | 2.3.1. **Trial being safe, low risk and not impacting standard care** could make one feel like there is 'nothing to lose' |  |  |  |  |  |  |  |  |  |  | **0** |
|  |  | 2.3.2. **Regain control when feeling desperate** by thinking there is 'nothing to lose', especially for those with cancer diagnosis and pre-eclampsia |  |  |  |  |  |  |  |  |  |  | **0** |
| 3. The impact of potential outcomes to participate | 3.1. Personal benefits of trial participation | 3.1.1. **Potentially benefiting their circumstances** by receiving treatments or new alternative for standard care |  |  |  |  |  |  |  |  |  |  | **0** |
|  |  | 3.1.2. **Potential benefits from trial** by getting quicker access to services, better follow-up care, increased contact with physicians, and learn more about their condition |  |  |  |  |  |  |  |  |  |  | **0** |
|  |  | 3.1.3. **Hope of relief when out of options** for those managing their own symptoms |  |  |  |  |  |  |  |  |  |  | **0** |
|  | 3.2. Societal benefits of trial participation | 3.2.1. **Altruism** in different combinations might be a motivating factor |  |  |  |  |  |  |  |  |  |  | **0** |
|  |  | 3.2.2. **Conditional altruism** or altruism dependent on the trial having low risk and clear personal benefits, especially important for life-changing cases such as cancer patients |  |  |  |  |  |  |  |  |  |  | **0** |
|  |  | 3.2.3. **Moral duty or obligation** to 'give back', usually for religious reasons or as an atonement for previous wrong doings |  |  |  |  |  |  |  |  |  |  | **0** |
|  |  | 3.2.4. Genuine interest in **contributing to scientific knowledge and improved care** |  |  |  |  |  |  |  |  |  |  | **0** |
| **Total of carbon relevant factors** | | | **5** | **8** | **6** | **0** | **0** | **9** | **7** | **0** | **0** | **0** |  |
