# Supplementary material for: Designing greener participant-centred trials: an analysis of ‘carbon relevant’ factors within items that influence participants’ decisions about trial recruitment and retention
Source: Trials. 2024 Apr 15;25:260. doi: 10.1186/s13063-024-08083-z (PMC11017479; doi:10.1186/s13063-024-08083-z)
Supplement: Supplementary file 2 — Additional file 2: Table S2. Reported influences on participant retention mapped to carbon relevant factors. [file 13063_2024_8083_MOESM2_ESM.docx]

Supplementary Table 2 – Reported influences on participant retention mapped to carbon relevant factors

| **Retention theme** | | | **Carbon relevant factors** | | | | | | | | | |  |
| --- | --- | --- | --- | --- | --- | --- | --- | --- | --- | --- | --- | --- | --- |
| **Verbatim theme** | **Sub-theme** | **Individual item** | **1. Trial set up** | **2. CTU emissions** | **3. Meetings and travel** | **4. Interventions** | **5. Data collection and exchange** | **6. Trial supplies and equipment** | **7. Trial specific patient assessment** | **8. Samples** | **9. Laboratory** | **10. Analysis and trial close out** | **Total themes** |
| 1. Perceptions of current health state in relation to specific aspects of the trial | 1.1. Perceptions of being 'too well' for trial | 1.1.1. **Perceptions of recovery** as a reason to not continue trial medication and/or problem-solving treatment exercises, and completing and returning to follow-up outcomes assessment questionnaires |  |  |  |  |  |  |  |  |  |  | 0 |
|  |  | 1.1.2. **Not wanting to be reminded about health issues** participants consider to be over by continuing the intervention or follow-up procedures |  |  |  |  |  |  |  |  |  |  | 0 |
|  | 1.2. Lack of compatibility with personal sense of self | 1.2.1. Belief of being able to **self-manage or cope well** without engagement of trial support intervention |  |  |  |  |  |  |  |  |  |  | 0 |
|  |  | 1.2.2. Belief that they were **unfit** to participate in group exercise intervention |  |  |  |  |  |  |  |  |  |  | 0 |
|  |  | 1.2.3. Belief they had **adequately managed their own condition** without need of medication |  |  |  |  |  |  |  |  |  |  | 0 |
|  |  | 1.2.4. **Not accepted their diagnosis** among those newly diagnosed |  |  |  |  |  |  |  |  |  |  | 0 |
|  |  | 1.2.5. **Emotional response** when participants had feelings of the trial not fitting with their personal sense of self |  |  |  |  |  |  |  |  |  |  | 0 |
|  | 1.3. Being 'too ill' to engage appropriately with trial intervention | 1.3.1. **Feeling 'too ill'** such as feeling either too fragile or depressed, too manic, or too emotional/ stressed, found in interventions regarding mental health conditions and focusing in newly diagnosed with type 1 diabetes |  |  |  |  |  |  |  |  |  |  | 0 |
|  |  | 1.3.2. Could **act as a 'trigger'** in terms of aggravating anxiety symptoms |  |  |  |  |  |  |  |  |  |  | 0 |
| 2. The 'fit' of aspects of the trial with individual preferences of care and support | 2.1. Aspects of trial 'fitting' the individual participant's preferences | 2.1.1. The design of intervention was **not individualized or tailored** enough to be helpful |  |  |  | + |  |  |  |  |  |  | 1 |
|  |  | 2.1.2. Interventions being **too technical, too physically demanding and too intensive** |  |  |  | + |  |  |  |  |  |  | 1 |
|  |  | 2.1.3. Interventions being **too basic** |  |  |  |  |  |  |  |  |  |  | 0 |
|  |  | 2.1.4. **Unhappy or dissatisfied or 'not comfortable'** with the treatment they received |  |  |  |  |  |  |  |  |  |  | 0 |
| 3. The compatibility of aspects of trial processes with individual capabilities | 3.1. Trial intervention and individual capabilities | 3.1.1. **Attention problems and limited reading and writing skills**, during internet delivered cognitive behavioural therapy and non-response to follow-up questionnaires |  |  |  |  |  |  |  |  |  |  | 0 |
|  |  | 3.1.2. **Feeling unintelligent** due to their inability to understand |  |  |  |  |  |  |  |  |  |  | 0 |
|  |  | 3.1.3. **Communication and cultural issues** |  | + | + |  |  | + |  |  |  |  | 3 |
| 4. Concerns about experiences of the trial medication | 4.1. Concerns of side effects of medication | 4.1.1. **Drug not being properly tested/ licensed** |  |  |  |  |  |  |  |  |  |  | 0 |
|  |  | 4.1.2. Trial medication could **negatively interact with other prescribed medication** |  |  |  |  |  |  |  |  |  |  | 0 |
|  |  | 4.1.3. **Dislike of taking too much medication** |  |  |  |  |  |  |  |  |  |  | 0 |
|  |  | 4.1.4. Trial medication **tasted offensive** |  |  |  |  |  |  |  |  |  |  | 0 |
| 5. Considerations around the extent to which trial participation could be appropriately accommodated into their broader lives | 5.1. Correlation with trial participation and participants' broader lives | 5.1.1. **Priority of other life 'events' and daily routines** such as work and family, exams, moving etc. |  |  |  |  |  |  |  |  |  |  | 0 |
|  |  | 5.1.2. **Lack of support from family members** |  |  |  |  |  |  |  |  |  |  | 0 |
|  |  | **5.1.3. 'Laziness' and 'forgetfulness'** |  |  |  |  |  |  |  |  |  |  | 0 |
| **Total carbon relevant factors** | | | 0 | 1 | 1 | 2 | 0 | 1 | 0 | 0 | 0 | 0 |  |
